# The Transcription Factor T-bet Regulates Intestinal Inflammation Mediated by Interleukin-7 Receptor^+^ Innate Lymphoid Cells

**DOI:** 10.1016/j.immuni.2012.09.008

**Published:** 2012-10-19

**Authors:** Nick Powell, Alan W. Walker, Emilie Stolarczyk, James B. Canavan, M. Refik Gökmen, Ellen Marks, Ian Jackson, Ahmed Hashim, Michael A. Curtis, Richard G. Jenner, Jane K. Howard, Julian Parkhill, Thomas T. MacDonald, Graham M. Lord

**Affiliations:** 1 Department of Experimental Immunobiology, Division of Transplantation Immunology and Mucosal Biology, King’s College London, London SE1 9RT, UK; 2 Centre for Immunology and Infectious Disease, Blizard Institute of Cell and Molecular Science, Barts & The London School of Medicine and Dentistry, London E1 2AT, UK; 3 National Institute for Health Research Biomedical Research Centre at Guy’s & St Thomas’ National Health Service Foundation Trust and King’s College London, London SE1 9RT, UK; 4 Pathogen Genomics Group, Wellcome Trust Sanger Institute, Wellcome Trust Genome Campus, Cambridgeshire CB10 1SA, UK; 5 Division of Diabetes and Nutritional Sciences, King’s College London, London SE1 9NH, UK; 6 Division of Infection and Immunity and UCL Cancer Institute, University College London, London WC1E 6BT, UK

## Abstract

Mice lacking the transcription factor T-bet in the innate immune system develop microbiota-dependent colitis. Here, we show that interleukin-17A (IL-17A)-producing IL-7Rα^+^ innate lymphoid cells (ILCs) were potent promoters of disease in *Tbx21*^−/−^*Rag2*^−/−^ ulcerative colitis (TRUC) mice. TNF-α produced by CD103^−^CD11b^+^ dendritic cells synergized with IL-23 to drive IL-17A production by ILCs, demonstrating a previously unrecognized layer of cellular crosstalk between dendritic cells and ILCs. We have identified *Helicobacter typhlonius* as a key disease trigger driving excess TNF-α production and promoting colitis in TRUC mice. Crucially, T-bet also suppressed the expression of IL-7R, a key molecule involved in controlling intestinal ILC homeostasis. The importance of IL-7R signaling in TRUC disease was highlighted by the dramatic reduction in intestinal ILCs and attenuated colitis following IL-7R blockade. Taken together, these data demonstrate the mechanism by which T-bet regulates the complex interplay between mucosal dendritic cells, ILCs, and the intestinal microbiota.

## Highlights

► Chronic colitis in TRUC mice was mediated by IL-17-producing innate lymphoid cells ► TNF-α synergized with IL-23 to induce innate IL-17 production ► *Helicobacter typhlonius* triggered intestinal pathology in TRUC mice ► T-bet regulated IL-7R transcription, a key checkpoint in intestinal ILC homeostasis

## Introduction

Interactions between the innate immune system and the intestinal microbiota play an important role in the maintenance of mucosal homeostasis. Genetic variation in innate immune components, such as pattern recognition receptors, is associated with Crohn’s disease (Barrett et al., 2008) and alters the susceptibility of mice to experimental inflammatory bowel disease (IBD) (Araki et al., 2005; Rakoff-Nahoum et al., 2004; Vijay-Kumar et al., 2007). Innate immune pathways can also drive gut inflammation, and numerous models of IBD are well characterized in mice lacking adaptive immunity (Buonocore et al., 2010; Kim et al., 2006; Li et al., 1998; Uhlig et al., 2006). Accordingly, there is considerable interest in understanding the mechanisms controlling innate immune activation in the gut.

T-bet, a T-box-family transcription factor, has emerged as a critical regulator of intestinal homeostasis and innate immunity, and mice lacking T-bet in the innate immune compartment spontaneously develop IBD (Garrett et al., 2007). The intestinal microbiota play a crucial role in TRUC (*Tbx21*^*−/−*^*Rag2*^*−/−*^ *u*lcerative *c*olitis) disease. IBD in this model resolves following treatment with antibiotics (Garrett et al., 2010; Garrett et al., 2007) and probiotics (Veiga et al., 2010) and does not occur when mice are raised in a germ-free environment (Garrett et al., 2010). Low-grade intestinal inflammation is also communicable to T-bet-sufficient mice, consistent with the notion that a colitogenic community of intestinal microbes triggers disease. Bacteria such as *Klebsiella pneumoniae* and *Proteus mirabilis* cultured from the feces of TRUC mice correlate with colitis but may not be causative (Garrett et al., 2010), given that colitis per se disrupts intestinal microbial ecology, resulting in nonspecific expansion of *Enterobacteriaceae* (Lupp et al., 2007; Stecher et al., 2007).

Importantly, the innate immune mechanisms responsible for maintaining chronic TRUC disease are not resolved. Early stages of disease are characterized by dysregulated TNF-α expression by colonic dendritic cells (DCs), and disease can be ameliorated by neutralizing TNF-α antibodies. However, beyond 12 weeks of age, TNF-α antibodies are ineffective (Garrett et al., 2009). The innate immune inflammatory pathways responsible for chronic colitis in TRUC mice are of interest because later stages of disease recapitulate some aspects of human ulcerative colitis (UC), with the proximal extension of inflammation, the development of fulminant colitis, and neoplasia (Garrett et al., 2007; Garrett et al., 2009).

Recently, the repertoire of cells that produce key cytokines implicated in IBD pathogenesis, such as interferon-γ (IFN-γ) and interleukin-17A (IL-17A), has been extended to include a population of cells termed innate lymphoid cells (ILCs) (Buonocore et al., 2010; Takatori et al., 2009). We hypothesized that ILCs would be responsible for driving fulminant TRUC disease and that T-bet would play a central role in regulating the pathogenicity of these cells.

## Results

### Chronic TRUC Disease Is ILC Dependent

**Figure 1:**
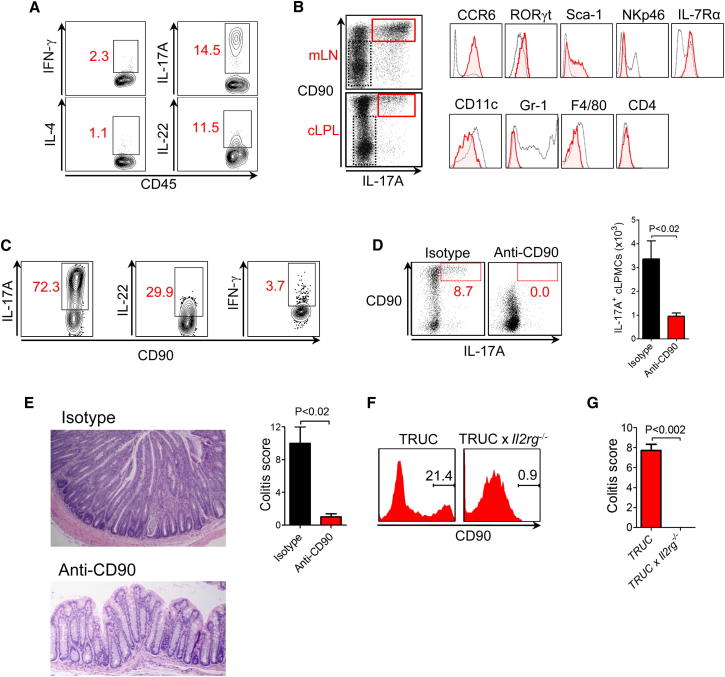
Chronic TRUC IBD Is Dependent on CD90^+^RORγt^+^CCR6^+^IL-7R^+^ ILCs (A) Intracellular cytokine expression by live, CD45^+^ cLP cells from TRUC mice following stimulation with PMA and ionomycin. (B) Phenotype of live, CD45^+^CD90^hi^IL-17A^+^ cells (red) in comparison with CD90^−^IL-17A^−^ cells (black) from the mLN and cLP of TRUC mice following stimulation with PMA and ionomycin. (C) Intracellular cytokine expression by lineage^−^ (CD11c^−^, NKp46^−^, Gr-1^−^) CD90^hi^ ILCs from mLN of TRUC mice. Cells were stimulated with PMA and ionomycin. (D) IL-17A and CD90 expression in live, CD45^+^ cLP cells following in vivo administration of anti-CD90 or control antibody to TRUC mice (left panel). Cells were stimulated with PMA and ionomycin. Right panel shows absolute numbers of IL-17A-producing cells in the cLP of these TRUC mice following CD90 depletion (n = 4) or control mAb treatment (n = 5). Results show mean, and error bars represent SEM. Also see Figure S1A. (E) Colon micrographs and colitis scores following depleting anti-CD90 treatment in TRUC mice. Results show mean, and error bars represent SEM. Other clinical features are shown in Figure S1B. (F) Flow cytometry histogram showing the % CD90^hi^ cells in the cLP of TRUC and TRUC × *Il2rg*^*−/−*^ mice. Also see Figure S1C. (G) Colitis scores of TRUC (n = 14) and TRUC × *Il2rg*^*−/−*^ (n = 5) mice. Results show mean, and error bars represent SEM. Other clinical features are shown in Figure S1D.

First, we tested the hypothesis that ILCs contribute to the pathogenesis of IBD in TRUC mice. We investigated the immune response in TRUC mice aged >12 weeks, an age at which TNF-α blockade loses efficacy and severe colitis emerges. We specifically looked for expression of IFN-γ, IL-17A, and IL-22 cytokines produced by innate immune cells, including ILCs. Activation of unfractionated colonic lamina propria leukocytes (cLPLs) from TRUC mice induced many IL-17A-expressing CD45^+^ immune cells. However, few IFN-γ- or IL-4-expressing cells were seen (Figure 1A). In TRUC mice, the majority of IL-17A-expressing cells were CD90^hi^, CCR6^+^, RORγt^+^, Sca-1^+^, and IL-7Rα^+^ (Figure 1B), consistent with the phenotype of ILCs (Buonocore et al., 2010; Sonnenberg et al., 2011). Intestinal IL-17A^+^CD90^+^ cells were CD4^−^, NKp46^−^, CD11c^−^, and Gr-1^−^. Notably, the cytokine response of CD90^+^ ILCs from TRUC mice was dominated by the expression of IL-17A and, to a lesser extent, IL-22; however, IFN-γ-expressing cells were conspicuously infrequent (Figure 1C).

For testing the functional significance of ILCs in chronic TRUC IBD, a depleting CD90 monoclonal antibody (mAb) was administered to TRUC mice. In contrast to effects on isotype-control-treated mice, anti-CD90 substantially reduced the number of CD90^+^ cells and the number of IL-17A-producing cells in TRUC mice (Figure 1D; Figure S1A available online) and ameliorated disease, including significantly improving colitis scores (Figure 1E), reduced colon mass, and reduced spleen mass (Figure S1B). In contrast, mice treated with control antibody developed fulminant colitis and severe histological changes characteristic of chronic TRUC IBD, including epithelial hyperplasia, goblet cell depletion, crypt destruction, crypt abscess formation, and infiltration of the lamina propria with mononuclear cells and neutrophils (Figure 1E).

To further investigate the role of ILCs in TRUC IBD, we generated *Tbx21*^*−/−*^*Rag2*^*−/−*^*Il2rg*^*−/−*^ triply deficient mice that additionally lack the common γ chain cytokine receptor. *Il2rg*^*−/−*^ mice have >90% reduction in the number of ILCs in secondary lymphoid organs, such as the spleen (Kim et al., 2005). We reasoned that intestinal ILC homeostasis would also be dependent on this receptor and therefore might provide additional insights into the requirements for ILCs in TRUC disease. Crucially, *Tbx21*^*−/−*^*Rag2*^*−/−*^*Il2rg*^*−/−*^ mice had severely diminished numbers of intestinal ILCs, failed to develop colitis, and had undetectable levels of *Il17a* messenger RNA (mRNA) in the colon (Figures 1F, 1G, and S1C–S1E). Taken together, these data demonstrate that chronic TRUC IBD is dependent on ILCs and that the common γ chain cytokine receptor plays an important role in intestinal ILC homeostasis.

### Chronic TRUC IBD Is Dependent on the IL-23:IL-17 Axis

**Figure 2:**
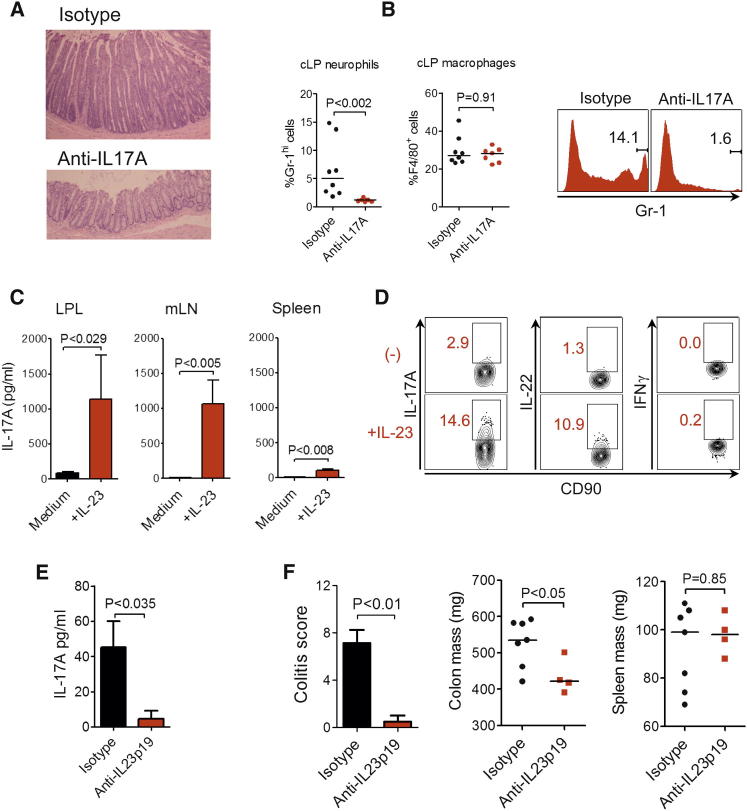
Chronic TRUC IBD Is Dependent on the IL-23:IL-17 Axis (A) Colon micrographs of TRUC mice following treatment with anti-IL17A (n = 7) or control antibody (n = 8). Also see Figure S2A for histology scores. (B) The proportion (%) of CD45^+^Gr-1^hi^CD11b^+^F4/80^−^ granulocytes and F4/80^+^CD11b^+^ macrophages in the cLP of TRUC mice treated with anti-IL17A or isotype. A representative flow cytometry histogram is also shown (right panel). (C) IL-17A concentration in the supernatants of cultured cLPLs, mLN cells, and splenocytes from TRUC mice, in the presence (+IL-23) or absence (medium alone) of recombinant IL-23, measured by ELISA. Results show mean, and error bars represent SEM. (D) Intracellular cytokine expression in lin^−^CD90^hi^ ILCs from mLN of TRUC mice following stimulation with IL-23. (E) IL-17A concentration in culture supernatants of explant organ culture of TRUC mice treated with anti-IL-23p19 or control antibody, measured by ELISA. Results show mean, and error bars represent SEM. (F) Colitis scores, colon mass, and spleen mass of TRUC mice treated with anti-IL-23p19 or control antibody. Results show mean, and error bars represent SEM.

In view of the dominant IL-17 response observed in chronic TRUC IBD, we investigated the role of the IL-23:IL-17 axis in this disease. We hypothesized that IL-17A blockade alone would be sufficient to ameliorate disease. In contrast to control antibody treatment, administration of a neutralizing IL-17A antibody markedly improved colitis (Figures 2A and S2A). Indeed, 71% (5/7) of anti-IL-17A-treated mice had complete resolution of colitis (colitis score = 0). Consistent with the biological activities of IL-17A, antibody blockade also prevented the accumulation of F4/80^−^CD11b^+^Gr-1^hi^ granulocytes infiltrating the colon (Figure 2B). However, the proportions of cLP F4/80^+^ macrophages were comparable in treated and untreated mice.

It has recently been reported that IL-23 also activates innate immunity, including driving innate IL-17 production (Takatori et al., 2009). Therefore, we investigated the role of IL-23 in TRUC IBD. IL-17A protein was inducible by IL-23 and was most pronounced in the colon and draining lymph nodes compared to the spleen of TRUC mice (Figure 2C). IL-23 induced the expression of IL-17A and, to a lesser extent, IL-22 by ILCs from TRUC mice but had little effect on IFN-γ production (Figure 2D). IL-23-induced ILC activation was also relevant in vivo. Administration of an IL-23p19 mAb significantly reduced IL-17A expression in ex vivo explant cultures (Figure 2E). Crucially, IL-23p19 neutralization also markedly improved colitis (Figure 2F). These data identify a central role for the IL-23:IL-17 axis in chronic intestinal inflammation in TRUC mice.

### *Helicobacter typhlonius* Is a Key Component of the Intestinal Microbiota Driving IBD in TRUC Mice

**Figure 3:**
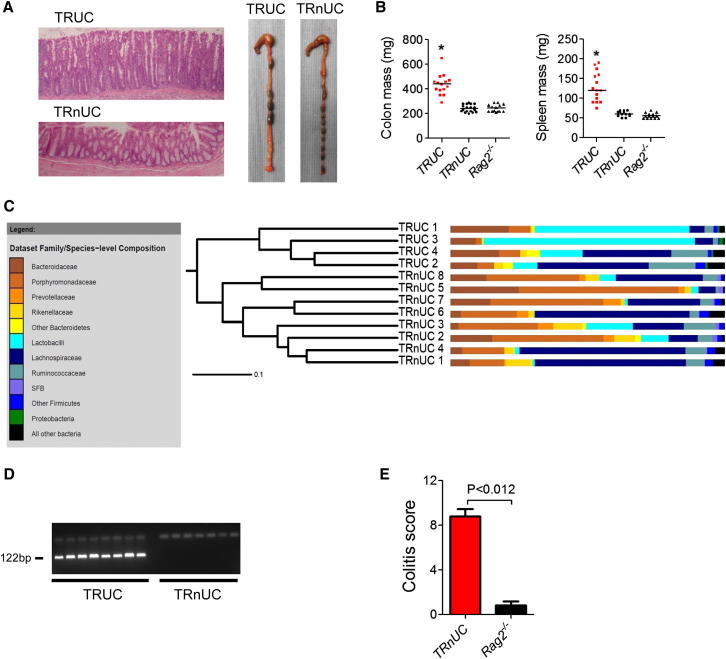
*H. typhlonius* Is a Key Component of the Intestinal Microbiota Driving IBD in TRUC Mice (A) Photomicrographs of the distal colon of 12-week-old TRUC mice with age matched, *Tbx21*^*−/−*^*Rag2*^*−/−*^ mice that did not develop spontaneous colitis (TRnUC) (left panel). Macroscopic appearances of the colon of 16-week-old TRUC and TRnUC mice (right panel). (B) Colon and spleen mass in 12- to 16-week-old TRUC, TRnUC, and *Rag2*^*−/−*^ mice. ^∗^p < 0.0001 (TRUC versus TRnUC or *Rag2*^*−/−*^). (C) Cluster dendrogram showing overall intestinal bacterial community membership clustering in TRUC and TRnUC mice. Families colored brown or yellow belong to the *Bacteroidetes* phylum; those in blue or purple belong to the *Firmicutes* phylum. SFB, segmented filamentous bacteria. Also see Figure S3B. (D) Agarose gel electrophoresis (2%) of PCR products following *H. typhlonius*-specific PCR performed on bacterial DNA isolated from fresh fecal samples from TRUC and TRnUC mice. The *H. typhlonius*-specific PCR product is 122 bp. (E) Colitis scores in TRnUC and *Rag2*^*−/−*^ mice following inoculation with pure cultures of *H. typhlonius* by oral gavage. Results show mean, and error bars represent SEM.

The composition of the intestinal microbiota is now recognized to profoundly impact intestinal immunity. Therefore, in order to better understand the immune pathways activated by the colitogenic microbiota colonizing TRUC mice, we sought to define which components of the microbiota were responsible for driving disease. Prior attempts to define causative bacteria have been hampered by a lack of appropriate control animals. However, we rederived a novel colony of *Tbx21*^*−/−*^*Rag2*^*−/−*^ mice that, unlike their isogenic TRUC counterparts, did not develop spontaneous colitis (Figures 3A and 3B). We termed these animals TRnUC (*Tbx21*^*−/−*^*Rag2*^*−/−*^ non-UC) mice. To identify potentially causative bacterial species, we compared the intestinal bacterial communities of TRUC and TRnUC mice by sequencing bacterial 16S ribosomal RNA (rRNA) genes. Individual samples shared broad similarities in community structure with other members of the same colony but were distinct from mice in the other colony (Figure 3C). As expected, all samples were dominated at the phylum level by *Firmicutes*, *Bacteroidetes*, *Proteobacteria*, *Actinobacteria*, and *Deferribacteres* (Figure S3A). At the phylum level, notable differences included an increased abundance of sequences from the *Proteobacteria* phylum and an increased ratio of *Firmicutes* to *Bacteroidetes* in TRUC mice (Figure S3B). However, just 12 species-level operational taxonomic units (OTUs) were consistently present in all TRUC mice and always absent from TRnUC mice. One of these sequences matched with 100% identity to the proteobacterial species *Helicobacter typhlonius* (Table S1). Specific PCR and DNA sequencing of the amplicon confirmed that *H. typhlonius* colonization was confined to TRUC mice (Figures 3D and S3C). Crucially, inoculation of TRnUC mice with pure cultures of *H. typhlonius* by oral gavage triggered severe colitis that was histologically indistinguishable from TRUC IBD (Figure 3E). *H. typhlonius* and colitis could also be transmitted to TRnUC mice cohoused with TRUC mice (data not shown). These data are consistent with an important role for *H. typhlonius* in triggering disease in TRUC mice.

### T-bet Is Required for Optimal Expression of IFN-γ by Intestinal ILCs, and in Its Absence ILCs Selectively Express IL-17A

**Figure 4:**
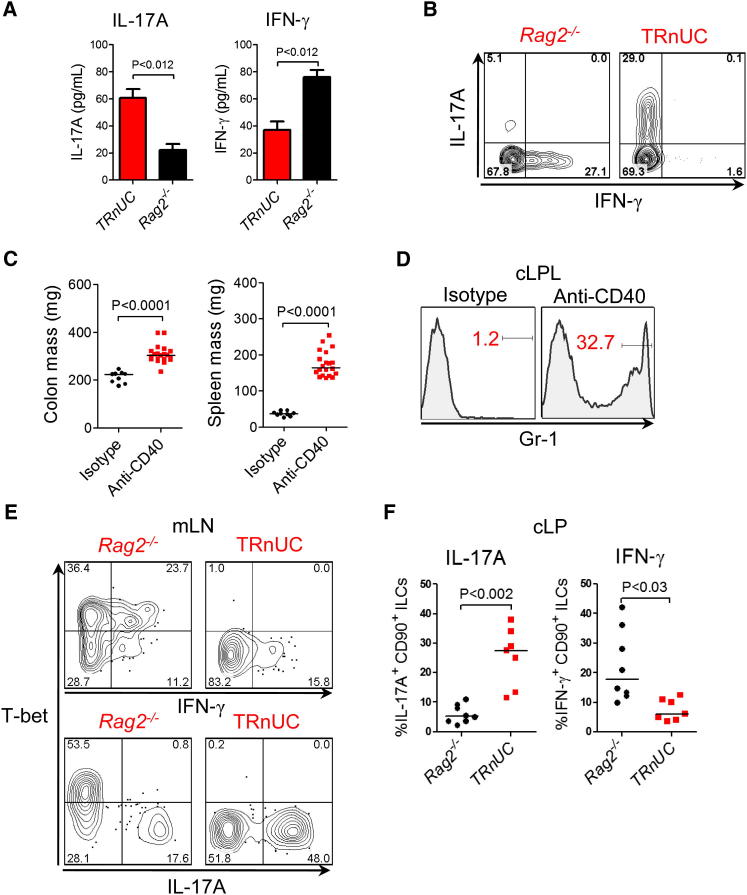
T-bet Is Required for Optimal Expression of IFN-γ by Intestinal ILCs, and in the Absence of T-bet ILCs Selectively Produced IL-17A (A) Cytokine concentrations in ex vivo explant cultures from *Rag2*^*−/−*^ (n = 5) and TRnUC (n = 5) mice following gavage with *H. typhlonius*, measured by ELISA. Results show mean, and error bars represent SEM. (B) Intracellular cytokine expression in live CD45^+^CD90^hi^ cLP ILCs from *Rag2*^*−/−*^ and TRnUC mice in *H. typhlonius*-associated colitis. Cells were stimulated with PMA and ionomycin. (C) Organ mass in *Rag2*^*−/−*^ mice following treatment with agonistic CD40 mAbs or control antibody. Also see Figure S4A for other clinical features. (D) Flow cytometry histogram demonstrating the proportion of Gr-1^hi^ granulocytes in the cLP of *Rag2*^*−/−*^ mice following anti-CD40 or control mAb administration. (E) Representative flow cytometry plot showing intracellular cytokine and T-bet expression in lin^−^CD90^+^ ILCs from mLN of *Rag2*^*−/−*^ and TRnUC mice following CD40 mAb treatment. (F) Proportions of IL-17A^+^ or IFN-γ^+^CD90^+^ ILCs in the cLP of *Rag2*^*−/−*^ or TRnUC mice following administration of anti-CD40.

The highly polarized IL-17A response and lack of significant IFN-γ expression observed in ILCs from TRUC mice led us to speculate that T-bet would be required for optimal production of IFN-γ by ILCs. To test this hypothesis, we compared the phenotypes of *Tbx21*^*+/+*^ and *Tbx21*^*−/−*^ intestinal ILCs in *H. typhlonius*-associated colitis in TRnUC and *Rag2*^*−/−*^ mice. Ex vivo explant cultures from TRnUC mice gavaged with *H. typhlonius* produced less IFN-γ and more IL-17A in comparison to *Rag2*^*−/−*^ mice (Figure 4A). Intracellular cytokine staining showed that ILCs from *Rag2*^*−/−*^ mice predominantly produced IFN-γ, whereas ILCs from TRnUC mice mostly produced IL-17A (Figure 4B). Similar results were also seen in another model of ILC-mediated intestinal inflammation induced by agonistic CD40 mAbs (Buonocore et al., 2010). Anti-CD40 induced wasting disease, splenomegaly, increased colonic mass, and infiltration of the colon with Gr-1^hi^ granulocytes (Figures 4C and S4A). Following disease induction, ILCs from *Rag2*^*−/−*^ mice expressed T-bet, which positively correlated with IFN-γ expression and inversely correlated with IL-17A expression. Conversely, IFN-γ expression was markedly diminished in ILCs from TRnUC mice, and instead these cells predominantly produced IL-17A (Figure 4D). Consistent with impaired IFN-γ production, wasting disease was delayed in T-bet-deficient mice; however, intestinal disease, as measured by increased colonic mass, occurred with comparable severity in this model (Figure S4B). These data indicate that T-bet is required for optimal expression of IFN-γ by intestinal ILC and that in the absence of T-bet these cells produce excess IL-17A.

### CD103^−^CD11b^+^ Colonic DCs Are Major Producers of TNF-α, which Potentiates IL-23-Induced Innate IL-17 Expression

**Figure 5:**
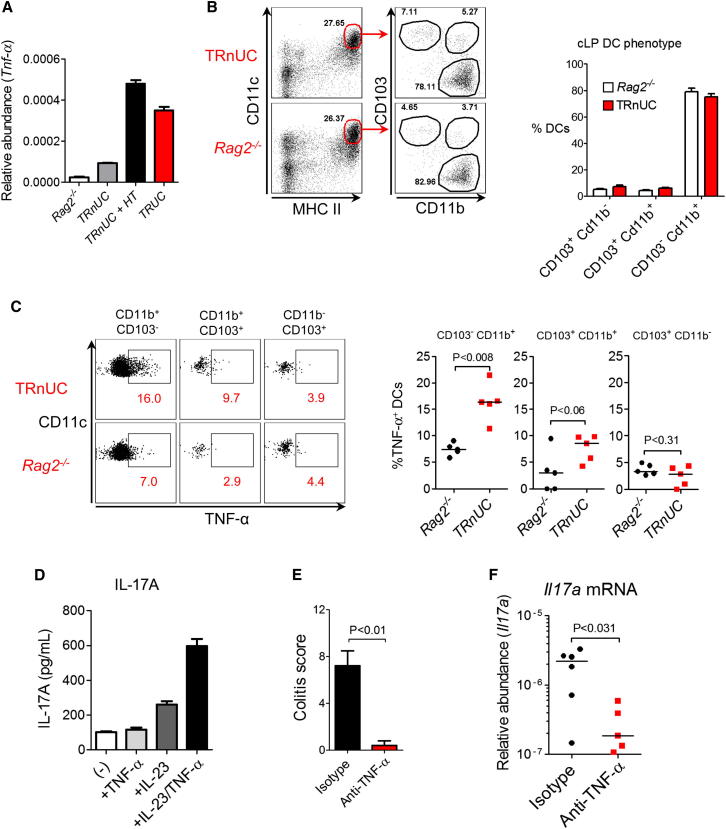
CD103^−^CD11b^+^ Colonic DCs Are Major Producers of TNF-α, which Potentiates IL-23-Induced Innate IL-17 Expression (A) Real-time PCR measuring *Tnfa* transcripts in the colons of *Rag2*^*−/−*^, TRnUC, TRnUC mice infected with *H. typhlonius* (+ *HT*), and TRUC mice. Results show mean, and error bars represent SEM. (B) DC subset frequency in the colon of TRnUC and *Rag2*^*−/−*^ mice following inoculation with *H. typhlonius*. Results show mean, and error bars represent SEM. (C) Spontaneous intracellular TNF-α production by different intestinal DC populations from *Rag2*^*−/−*^ and TRnUC mice following inoculation with *H. typhlonius*, showing representative flow cytometry plots (left panel) and statistical analysis (n = 5 per group) (right panel). (D) IL-17A production by unfractionated mLN cells from TRUC mice following incubation with the stimuli described. (-) indicates unstimulated cells. Results show mean, and error bars represent SEM. (E) Colitis scores in TRnUC mice inoculated with *H. typhlonius* following treatment with a blocking TNF-α mAb or control antibody. Results show mean, and error bars represent SEM. (F) Real-time PCR quantifying *Il17a* transcripts following treatment with a neutralizing TNF-α mAb or control isotype.

Deregulated TNF-α production by colonic CD11c^+^ DCs is a hallmark feature of TRUC IBD (Garrett et al., 2007; Garrett et al., 2009). Therefore, we investigated whether *H. typhlonius* was capable of inducing TNF-α expression by colonic DCs in TRnUC mice and also how this might impact innate IL-17 production. Oral inoculation with *H. typhlonius* resulted in increased abundance of *Tnfa* transcripts in the colon of TRnUC mice to levels similar to those observed in TRUC mice (Figure 5A). CD11c^hi^ major histocompatibility complex class II^+^ intestinal DCs can be divided into subsets based on surface expression of CD103 and CD11b (Varol et al., 2009). The frequency of each colonic DC subset was comparable in *Rag2*^*−/−*^ and TRnUC mice (Figure 5B). CD103^−^CD11b^+^ DCs were the principal subset responsible for TNF-α production and were also the most frequent colonic DC subset. Notably, in the absence of T-bet, TNF-α expression by CD103^−^CD11b^+^ DCs was enhanced 2 to 3 fold (Figure 5C). We sought to determine how enhanced TNF-α production by CD103^−^CD11b^+^ intestinal DCs might impact ILC activation in TRUC IBD. Although on its own, recombinant TNF-α was unable to induce innate IL-17A production by unfractionated mesenteric lymph node (mLN) cells from TRUC mice, it markedly potentiated IL-23-induced IL-17A production (Figure 5D). We also investigated the functional significance of TNF-α-potentiated innate IL-17 expression in vivo. As well as ameliorating *H. typhlonius*-induced colitis in TRnUC mice (Figure 5E), TNF-α blockade also substantially reduced colonic *Il17a* transcripts, consistent with the possibility that TNF-α-augmented IL-17A production is physiologically relevant in ILC-mediated colitis (Figure 5F).

### TRUC IBD Is Critically Dependent on IL-7 Signaling

**Figure 6:**
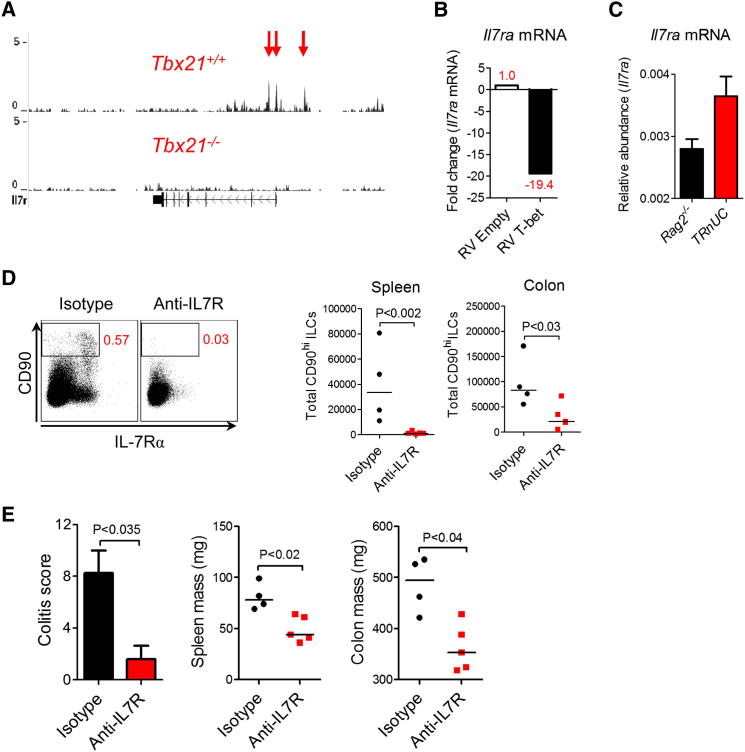
TRUC Disease Is Critically Dependent on IL-7R Signaling (A) UCSC Genome Browser image is shown for T-bet binding at the *il7ra* locus in *Tbx21*^*+/+*^ versus *Tbx21*^*−/−*^ CD4^+^ Th1 cells following stimulation with PMA and ionomycin for 4 hr. Chromatin was immunoprecipitated, crosslinked, and prepared as described previously (Jenner et al., 2009). Samples were sequenced with an Illumina Genome Analyzer II-x. The vertical axis depicts the number of tags per million total sequences, with the genomic location running along the horizontal axis. The direction of transcription is indicated by arrows. Red arrows depict three sites of T-bet binding at the *Il7ra* locus. (B) Relative abundance of *Il7ra* mRNA in *Tbx21*^*−/−*^*Ifng*^*−/−*^ CD4^+^ T cells following retroviral transduction with empty vector (RV) or T-bet (T-bet RV). Cells were activated with PMA and ionomycin. (C) Relative abundance of *Il7ra* mRNA in fluorescence-activated cell-sorted *Tbx21*^*−/−*^ and *Tbx21*^*+/+*^ CD90^hi^ ILCs isolated from mLN of TRnUC and *Rag2*^*−/−*^ mice following induction of disease with anti-CD40. Results show mean, and error bars represent SEM. (D) Representative flow cytometry plot of splenic CD90^+^IL-7R^+^ ILCs (left panel) and absolute numbers (right panel) of ILCs in the spleen and colon of TRUC mice, following treatment with anti-IL7R or control antibody. (E) Colitis score (left panel), spleen mass (middle panel), and colon mass (right panel) in TRUC mice following treatment with anti-IL-7R or control antibody. Results show mean, and error bars represent SEM.

We next determined whether T-bet might regulate other genes involved in ILC biology. The majority of ILCs in TRUC disease and other ILC-associated diseases (Buonocore et al., 2010; Sonnenberg et al., 2011) express IL-7 receptor (IL-7R), which appears to play an important role in the homeostasis of several immune cell populations (Crellin et al., 2010; Satoh-Takayama et al., 2010; Schmutz et al., 2009). Indeed, intestinal ILCs are reported to be absent or reduced in *Il7*^−/−^ mice (Vonarbourg et al., 2010). IL-7R is also a member of the common γ chain cytokine receptor family, which plays an indispensible role in intestinal ILC homeostasis, as we have already demonstrated (Figures 1F and 1G). Although ILCs are too infrequent to perform chromatin immunoprecipitation sequencing, we were able show that T-bet binds at the *Il7ra* locus in CD4^+^ T cells. T-bet binding was enriched at the transcriptional start site of the *Il7ra* gene in a region highly conserved in mammals (Figure 6A). Next, we asked whether T-bet was a transcriptional activator or repressor of the *Il7ra* gene. Retroviral transduction of T-bet into CD4^+^ T cells from *Tbx21*^*−/−*^*Ifng*^*−/−*^ mice resulted in a marked reduction in *Il7ra* mRNA expression (Figure 6B). Crucially, *Il7ra* transcripts were reduced in T-bet-expressing ILCs from *Rag2*^*−/−*^ mice in comparison with T-bet-deficient ILCs from TRnUC mice (Figure 6C), consistent with the possibility that T-bet represses IL-7Rα expression in ILCs in a physiologically relevant disease setting. IL-7R signaling was also functionally important in intestinal ILC homeostasis and TRUC disease. IL-7R blockade significantly reduced ILC numbers in TRUC mice, although ILCs in secondary lymphoid tissue appeared to be more sensitive to blockade than intestinal ILCs, given that CD90^hi^IL-7Rα^+^ ILCs were almost completely eliminated from the spleen, whereas ILC numbers in the colon were diminished 3 to 4 fold following anti-IL-7R treatment (Figure 6D). IL-7R blockade also improved disease parameters (Figure 6E), demonstrating that IL-7R signaling plays an important role in intestinal ILC homeostasis and controls ILC-mediated mucosal pathology.

## Discussion

Our results shed new light on the TRUC model of IBD and provide novel insight into the regulation of ILC-mediated mucosal pathology. We have demonstrated that ILCs potently promoted colitis in TRUC mice. Excess TNF-α produced by CD103^−^CD11b^+^ colonic DCs synergized with IL-23 to induce IL-17A expression by ILCs, demonstrating a previously unrecognized layer of crosstalk between DCs and ILCs. We have also identified a constituent of the TRUC intestinal microbiota that augmented colonic TNF-α production and triggered severe colitis in rederived, otherwise healthy, isogenic *Tbx21*^*−/−*^*Rag2*^*−/−*^ hosts. Lastly, we have shown that T-bet repressed the expression of IL-7R, signaling through which is critical for intestinal ILC homeostasis and TRUC IBD. Together, these data demonstrate that T-bet controls a transcriptional program in the intestinal innate immune system that profoundly impacts the balance between the maintenance or loss of intestinal homeostasis. These data also highlight an important aspect of transcription factor biology, wherein a single transcription factor simultaneously regulates multiple target genes involved in a particular immune pathway. In a similar way, we have previously shown that T-bet binds at the promoter of >800 protein-encoding genes in T helper 1 (Th1) cells, which results in the activation of numerous key genes involved in the Th1 cell response (Jenner et al., 2009), ensuring that an efficient, coordinated and appropriately polarized response ensues (Powell et al., 2010).

These new insights necessitate an update in our understanding of the TRUC model of IBD. We have shown that the key cellular mediators of chronic intestinal inflammation in TRUC mice were CD90^hi^RORγt^+^CCR6^+^IL-7Rα^+^ ILCs. ILCs were the primary source of IL-17A in this disease, and ILC depletion reversed colitis. Strikingly, in the absence of T-bet, intestinal ILCs produced minimal amounts of IFN-γ and instead selectively expressed IL-17A. These data suggest that pronounced IL-17 production by ILCs, even when unaccompanied by substantial IFN-γ coexpression, is sufficient to induce severe intestinal pathology. These data also indicate that T-bet is necessary for optimal IFN-γ expression by intestinal ILCs. Diminished innate production of IFN-γ observed in T-bet-deficient hosts probably accounts for the delayed wasting disease observed in anti-CD40-induced disease, consistent with previous reports showing that systemic disease in this model is mediated by the IL-12/IFN-γ pathway (Uhlig et al., 2006). These data also imply that T-bet impacts innate immunity in a tissue-specific manner and that intestinal rather than systemic disease is favored in its absence. In contrast to other studies showing that ILC-mediated intestinal pathology requires dual blockade of IL-17 and IFN-γ to abrogate disease (Buonocore et al., 2010), in TRUC IBD IL-17A blockade alone reversed disease. It is possible that IL-17A blockade alone would not fully prevent ILC-mediated pathology in T-bet-sufficient hosts because, unlike TRUC mice, these animals can still mount effective IFN-γ responses. These observations offer potentially relevant insights into clinical trials evaluating cytokine blockade in human IBD. Antibody blockade of IFN-γ is largely disappointing in Crohn’s disease (Reinisch et al., 2010), and preliminary data appear to show that anti-IL-17A therapy lacks efficacy or even exacerbates Crohn’s disease (W. Hueber et al., 2011, J. Crohns Colitis, abstract). Although redundancy and plasticity of cytokine responses potentially account for the lack of efficacy observed following anti-IL-17 or anti-IFN-γ monotherapy, it is now appreciated that IBD is heterogeneous in terms of clinical phenotype, mucosal immune response, and genetic risk. In the future it is conceivable that IBD patients will be stratified according to their genetic profile or mucosal immune response phenotype in order to better guide selective cytokine blockade in individual patients. However, blockade of IL-12p40, which is required for biological activity of both IL-12 and IL-23 and hence is a common upstream signal for both IL-17A and IFN-γ production, shows therapeutic promise in IBD (Mannon et al., 2004).

We also identified *H. typhlonius* as a key colitogenic component of the TRUC intestinal microbiota. This bacterium was ever present in the intestinal microbiota of diseased TRUC mice and was consistently absent from rederived, disease-free *Tbx21*^*−/−*^*Rag2*^*−/−*^ mice. Oral inoculation with pure cultures of *H. typhlonius* was also sufficient to trigger disease. *H. typhlonius* induced markedly more severe IBD in T-bet-deficient hosts, confirming that T-bet deficiency confers heightened susceptibility to IBD following exposure to particular intestinal microbes. *H. typhlonius* is a Gram-negative, microaerophilic, urease-negative, spiral-rod-shaped bacterium that is closely related to *Helicobacter hepaticus* (Franklin et al., 2001). *H. typhlonius* was first identified in the colon of *Il10*^*−/−*^ mice that also develop microbiota-dependent IBD (Fox et al., 1999; Franklin et al., 2001). Evidence is mounting in humans to suggest that IBD may also be linked to expansion of colitogenic microbes and/or contraction of protective bacteria. Adherent-invasive *Escherichia coli* are associated with IBD and have been implicated in disease pathogenesis (Chassaing et al., 2011). Conversely, intestinal colonization with *Faecalibacterium prausnitzii*, a bacterium with anti-inflammatory properties (Sokol et al., 2008), is negatively correlated with Crohn’s disease (Joossens et al., 2011) and UC (Sokol et al., 2009). The TRUC model of IBD therefore highlights how the genetic composition of the host shapes interactions with the intestinal microbiota and impacts colitis susceptibility.

We have also shown that colonic TNF-α, which was primarily produced by CD103^−^CD11b^+^ DCs, was augmented in the absence of T-bet. Although by itself TNF-α did not appear to induce marked innate IL-17 production, in combination with IL-23 it proved to be a potent stimulus for IL-17 production. These data indicate that crosstalk between DCs and ILCs may be important in the regulation of intestinal homeostasis and that T-bet plays a crucial role in regulating this interaction. In the absence of T-bet, CD103^−^CD11b^+^ DCs overproduced TNF-α, which synergized with IL-23 to induce IL-17A production by IL-7R^+^ ILCs. Therefore, in the appropriate microbiological context, T-bet deficiency favors excess intestinal production of TNF-α and IL-17A, which appears to be a potently colitogenic cytokine milieu.

We have also shown that IL-7R blockade attenuated ILC-mediated IBD. Similar to the situation in *Il2rg*^*−/−*^ mice, which are unable to signal through IL-7R, specific IL-7R blockade significantly diminished colonic ILCs and suppressed colitis. Interestingly, unlike splenic ILCs that were almost completely eliminated, colonic ILCs were less prone to depletion by IL-7R blockade, indicating that additional IL-7-independent ILC survival signals may exist in the colon or in inflamed tissue. The importance of IL-7R signaling in IBD is also implied by recent data identifying SNPs at the *IL7R* locus in patients with UC (Anderson et al., 2011). We have shown that T-bet bound at the *Il7ra* promoter and, when overexpressed, reduced *Il7ra* mRNA expression 20 fold in T cells in an IFN-γ-independent manner, consistent with the possibility that T-bet is a transcriptional repressor of the *Il7ra* locus. In keeping with this, *Il7ra* mRNA was increased in ILCs from T-bet-deficient hosts.

In conclusion, these results demonstrate that T-bet is a crucial gatekeeper of innate inflammatory pathways at the intestinal barrier surfaces, where it regulates the balance between mucosal homeostasis and inflammation. In the absence of T-bet, particular microbes trigger overproduction of colonic TNF-α, which drives chronic colitis that is mediated by innate lymphoid cells preferentially producing IL-17A.

## Experimental Procedures

### Animal Husbandry

Balb/C *Tbx21*^*−/−*^ (Jackson Labs), *Rag2*^*−/−*^ (Jackson Labs), *Rag2*^*−/−*^*Il2rg*^*−/−*^ (Taconic), and *Ifng*^*−/−*^ (Jackson Labs) mice were sourced commercially. A colony of colitis-free TRnUC mice was generated and remotely maintained at geographically distinct isolators from the TRUC colony that was descendant from the originally described TRUC mice (Garrett et al., 2007). All animal experiments were conducted in accredited facilities in accordance with the UK Animals (Scientific Procedures) Act 1986 (Home Office license number PPL 70/6792).

### Isolation of cLPLs

Colons were cut longitudinally, fecal material was removed, and colons were washed with PBS. Colons were then cut in to 5 mm segments, and the epithelium was removed through incubation with Hank’s balanced salt solution (HBSS) without Mg^2+^ or Ca^2+^ (Invitrogen), supplemented with 10% fetal calf serum (FCS Gold, PAA Laboratories), 10 mM HEPES (Fisher Scientific), 100 IU/ml penicillin, and 100 μg/ml streptomycin (Invitrogen) and EDTA (5 mM). Tissue was then mechanically disrupted using GentleMACS (Miltenyi Biotec), followed by collagenase digestion through incubation of the crude cell mixture in RPMI-1640 medium (PAA Laboratories) supplemented with 25 μg/ml collagenase D (Roche), 10 μg/ml DNase I (Roche), and 1.5mg/ml dispase (Roche) for 45 min in a shaking water bath (37°C). Cells were resuspended in 40% Percoll (GE Healthcare) and layered on 80% Percoll prior to centrifugation, and the cLPL-enriched population was harvested.

### Histology

One centimeter segments of colon were fixed in 10% paraformaldehyde and embedded in paraffin blocks. Five micrometer sections were stained with hematoxylin and eosin. For immunofluorescence, colon tissue was removed and snap frozen in Jung tissue-freezing medium (Leica Microsystems). Seven micrometer cryostat sections were fixed in acetone, blocked (20% normal horse serum, PAA Laboratories), and incubated with fluorescein-isothiocyanate-conjugated anti-CD90.2 (eBioscience). Nuclei were counterstained with 1 μg/ml DAPI (Invitrogen). Colitis scores comprising epithelial hyperplasia (0–3), epithelial injury (0–3), polymorphonuclear infiltrate (0–3), and mononuclear infiltrate (0–3) were reported in a blinded fashion (T.T.M.) as described previously (Garrett et al., 2007).

### Flow Cytometry

Intracellular cytokine expression was measured after cells were stimulated with IL-23 (20 ng/ml) or with PMA (50 ng/ml,) and ionomycin (1 μg/ml, both Sigma-Aldrich) for 4 hr, with monensin (2 μM) added for the final 2 hr at 37°C. In order to measure spontaneous cytokine production by DCs, cells were incubated for 4 hr at 37°C in the presence of monensin without additional stimuli. Surface-staining antibodies were added together with LIVE/DEAD stain (Invitrogen) for 20 min at room temperature. Cells were fixed in 1% paraformaldehyde and permeabilized (eBioscience permeabilization buffer), and intracellular staining was performed for 30 min at 4°C. Antibodies were from eBioscience unless otherwise stated: α-CD45 (30-F11), α-CD4 (RM4.5), α-CD11b (M1/70), α-CD11c (N418), α-CD103 (2E7), α-F4/80 (BMP), α-CD90.2 (30H12), α-NKp46 (29A1.4), α-Gr-1 (RB6-8C5), α-RORγt (AFKJS-9), α-CCR6 (140706, R&D systems), α-Sca-1 (D7, Invitrogen), α-T-bet (eBio4B10), α-IFN-γ (XMG1.2), α-IL-17A (eBio17B7), α-IL-22 (IH8PWSR), α-TNF-α (MP6-XT22), α-IL-4 (11B11, BD PharMingen), and α-CD127 (A7R34).

### RNA Extraction and Quantitative PCR

RNA was extracted from colon segments or purified cells with Trizol reagent (Invitrogen) and complementary DNA (cDNA) generated with the cDNA synthesis kit (Bioline). Quantitative PCR was used to quantify mRNA transcripts using TaqMan gene-expression assays (Applied Biosystems, Warrington, UK). Gene expression was normalized to the expression of β-actin for generation of ΔCt values, and relative abundance was quantified with the 2^−ΔCt^ method.

### In Vivo Treatment of Mice

Antibody treatments comprised: 500 μg anti-TNF-α (clone XT3.11, days 0, 3, 6, 9, 12, 15, 18, and 21), 150 μg anti-IL23p19 (G23-8, eBioscience, days 0, 4, 8, 13, 16, and 20), 450 μg IL-17A mAb (17F3, days 0, 4, 8, 11, 15, 18, and 21), 1 mg CD90.2 mAb (30H12, days 0, 7, 14, 21, and 28), and 1 mg anti-IL-7R (A7R37, days 0, 3, 6, 9, 12, 15, 18, and 21). Control-isotype clones used were MOPC-21 (mouse immunoglobulin G1 [IgG1]), HRPN (rat IgG1), 2A3 (rat IgG2a), and LTF-2 (rat IgG2b). In the anti-CD40 model, 150 μg of mAb (clone FGK4.5) or control isotype was administered to 6- to 8-week-old mice. Monoclonal antibodies were purchased from Bio X Cell (West Lebanon, NH, USA) unless otherwise stated and were administered intraperitoneally.

### 454 Pyrosequencing Analysis of Intestinal Microbiota 16S rRNA Genes

DNA was extracted from frozen fecal pellets with the FastDNA SPIN Kit. 16S-rRNA-gene PCR amplicons were generated for Lib-L 454 Titanium sequencing with the use of barcoded primers targeting the V3–V5 regions of the 16S rRNA gene. The primers and barcode sequences used are given in Table S2. PCR products were generated with AccuPrime Taq DNA Polymerase High Fidelity (Invitrogen). PCR cycling conditions were as follows: 94°C for 2 min followed by 20 cycles of 94°C for 30 s, 53°C for 30 s, and 68°C for 2 min. PCR products were then quantified with a Qubit 2.0 Fluorometer (Invitrogen), and equimolar volumes of each were added to a master mix for sequencing.

Raw sequences were passed through the PyroTagger pipeline (Kunin et al., 2010), which filters poor-quality reads, clusters sequences into OTUs at 97% similarity, assigns taxonomic classifications to each OTU based on the Greengenes (DeSantis et al., 2006) and SILVA (Pruesse et al., 2007) databases, and trims them to a length of ∼400 bases. Further taxonomic classifications for each OTU were obtained with the mothur software package (Schloss et al., 2009) for classifying the sequences according to the Ribosomal Database Project (Cole et al., 2009) and SILVA (Pruesse et al., 2007) databases. After processing and subsequent manual removal of suspect or chimeric OTUs, 33,528 sequences remained, which were split into 256 OTUs overall. The median number of sequences per sample was 2,817 (range 1,781–3,884). OTUs that were significantly differentially abundant between the TRUC and two TRnUC mouse cohorts were identified with the Metastats program (Schloss et al., 2009; White et al., 2009).

### *Helicobacter* PCR

Bacterial DNA was extracted from feces with the QIAamp DNA stool minikit (QIAGEN). *H. typhlonius*-specific PCR was performed with the use of primer pairs that have been shown to amplify a 122 bp sequence of the 16S rRNA gene in *H. typhlonius*, but not other *Helicobacter* species (Feng et al., 2005) (5′-AGGGACTCTTAAATATGCTCCTAGAGT-3′ and 5′-ATTCATCGTGTTTGAATGCGTCAA-3′). The PCR conditions used were as follows: 95°C for 30 s, 55°C for 30 s, and 72°C for 30 s for 35 cycles. *Helicobacter*-genus-generic PCR was performed with the primer pair (5′-TATGACGGGTATCCGGC-3 and 5′-ATTCCACCTACCTCTCCCA-3′) (Beckwith et al., 1997). The 374 bp PCR product generated by genus-generic primers was recovered from acrylamide gel for subsequent DNA sequencing performed by an outside vendor (Charles River Laboratories, Wilmington, MA, USA).

### Bacterial Growth Conditions

*H. typhlonius* CCUG 48335 T was obtained from the culture collection of the University of Goteborg, Sweden (Franklin et al., 2001) and was grown and maintained either on blood agar plates (Oxoid, Hampshire, UK) containing 5% defibrinated horse blood (TCS Biosciences, Buckingham, UK) or in brain-heart infusion broth supplemented with 1% yeast extract and 5% horse serum. Cultures were incubated in an anaerobic atmosphere consisting of 80% N_2_, 10% H_2_, and 10% CO_2_ (Don Whitley Scientific, Shipley, UK). A total of 1–5 × 10^7^ organisms were gavaged into selected mice as described in the text.

### Cell Culture

Unfractionated splenocytes (2 × 10^6^/ml), mLN (1 × 10^5^/ml), and cLP cells (1 × 10^5^/ml) were cultured in RPMI-1640 medium (PAA Laboratories), supplemented with 10% FCS (PAA Laboratories), 50 μM 2-mercaptoethanol (Invitrogen), 2mM L-glutamine (Sigma-Aldrich), 1 mM sodium pyruvate (Invitrogen), 10 mM HEPES (Fisher Scientific), nonessential amino acids (Sigma-Aldrich), 100 IU/ml penicillin, and 100 μg/ml streptomycin (Invitrogen). Cells were cultured in medium alone or in the presence of 20ng/ml recombinant IL-23 and/or TNF-α (R&D Systems). Cytokine concentrations in culture supernatants were measured via ELISA (R&D Systems).

### Ex Vivo Organ Culture

Three-millimeter punch biopsies (Miltex) were used to acquire full thickness colonic biopsies. Three biopsies were cultured in 300 μl of RPMI supplemented with complete medium (as above) for 24 hr. Cytokine concentrations in culture supernatants were measured by ELISA (R&D Systems).

### Retroviral Transduction

CD4^+^ T cells from *Tbx21*^*−/−*^*Ifng*^*−/−*^ mice were cultured in Th0 conditions with IL-2 only (20 ng/ml, R&D Systems, Abingdon, UK) and transduced with either retrovirus encoding T-bet and green fluorescent protein (GFP) (RV-GFP-T-bet) or control retrovirus encoding GFP only (RV-GFP) as described previously (Szabo et al., 2000). Cells were sorted for GFP expression on day 5. Cells were stimulated for 4 hr with PMA and ionomycin, and RNA was extracted with Trizol reagent (Invitrogen).

### Statistical Analysis

Nonparametric data were analyzed with the Mann-Whitney U test using GraphPad Prism. Elsewhere, mean data are expressed with error bars denoting SEM.
