# Supplementary material for: The Transcription Factor T-bet Regulates Intestinal Inflammation Mediated by Interleukin-7 Receptor+ Innate Lymphoid Cells
Source: Immunity. 2012 Oct 19;37(4):674–84. doi: 10.1016/j.immuni.2012.09.008 (PMC3540260; doi:10.1016/j.immuni.2012.09.008)
Supplement: Document S1. Figures S1–S4 and Tables S1 and S2 [file mmc1.pdf]

Immunity, Volume *37*

Supplemental Information

The Transcription Factor T-bet Regulates Intestinal Inflammation Mediated by Interleukin-7 Receptor$^+$ Innate Lymphoid Cells

Nick Powell, Alan W. Walker, Emilie Stolarcyzk, James B. Canavan, M. Refik Gökmen, Ellen Marks, Ian Jackson, Ahmed Hashim, Mike A. Curtis, Richard G. Jenner, Jane K. Howard, Julian Parkhill, Thomas T. MacDonald, and Graham M. Lord

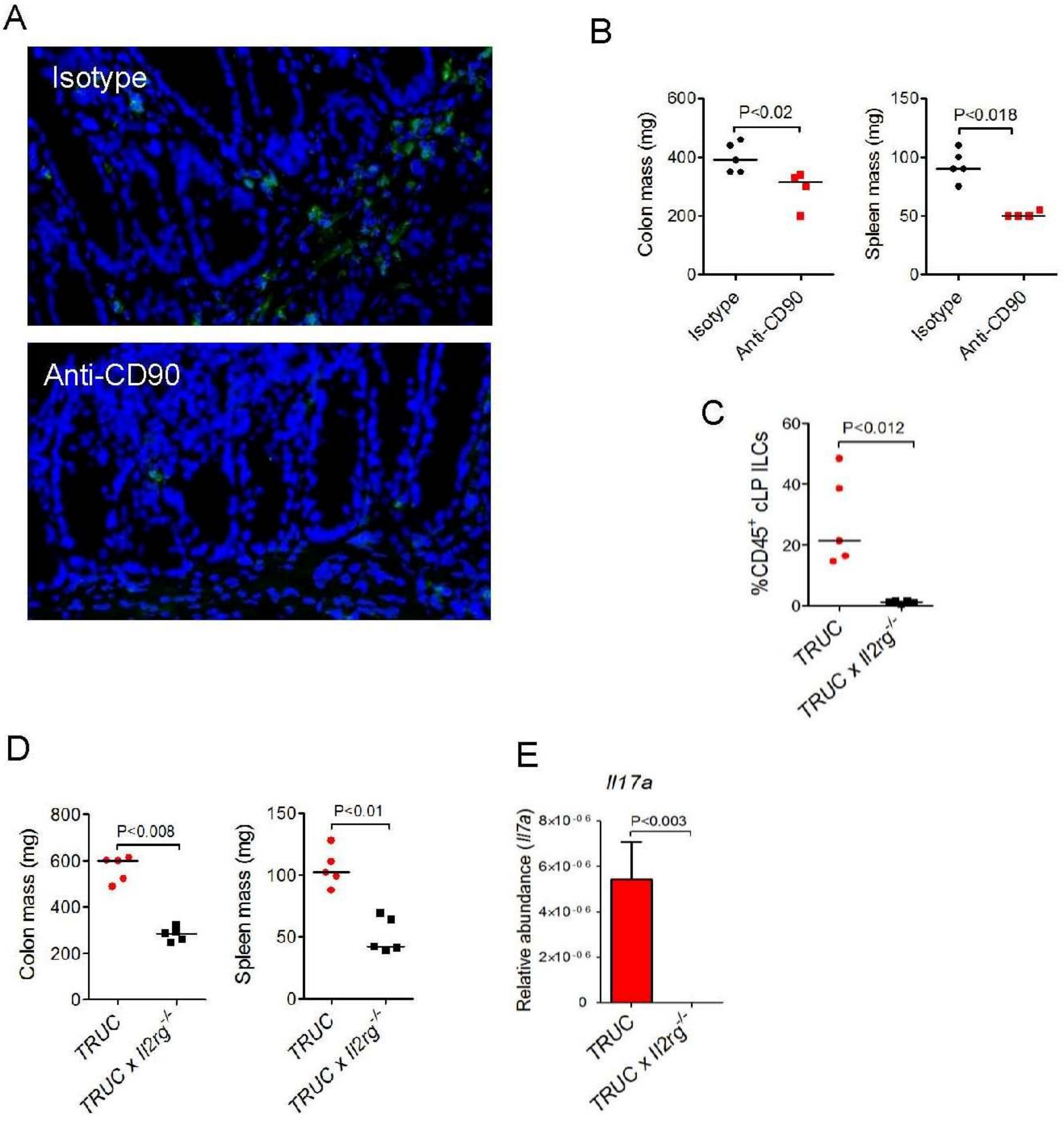

Figure S1. Chronic Intestinal Inflammation in TRUC Mice Is Dependent on Innate Lymphoid Cells

(A) Immunofluorescence (x20 magnification) staining for CD90 (green) in the cLP of TRUC mice treated with anti-CD90 or control antibody (IgG2b). Nuclei are counterstained with DAPI. (B) Colon mass and spleen mass in TRUC mice following treatment with depleting anti-CD90 mAb or control Isotype. (C) Proportion (%) of $CD90^{high}$ ILCs (as a proportion of live, $CD45^{+}$) cLP cells in TRUC (n=5) and *Tbx21*$^{-/-}$*Rag2*$^{-/-}$*Il2rg*$^{-/-}$ (n=5) mice. (D) Colon mass and spleen mass in TRUC (n=5) and *Tbx21*$^{-/-}$*Rag2*$^{-/-}$*Il2rg*$^{-/-}$ mice (n=5). (E) Real time PCR quantifying *Il17a* transcripts in the colon of TRUC (n=14) and *Tbx21*$^{-/-}$*Rag2*$^{-/-}$*Il2rg*$^{-/-}$ (n=5) mice. Results show mean, and error bars represent SEM.

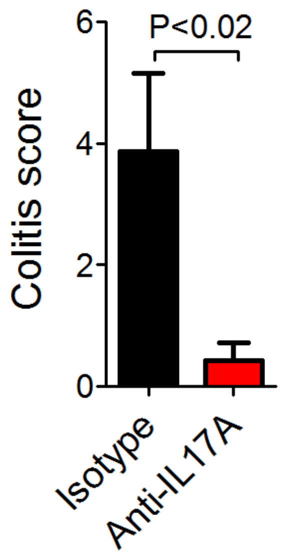

Figure S2. IL-17A Blockade Significantly Reduced Colitis Histology Scores in TRUC Mice

(A) Colitis scores in TRUC mice treated with anti-IL-17A (n=7) or isotype control mAb (n=8). Results show mean, and error bars represent SEM.

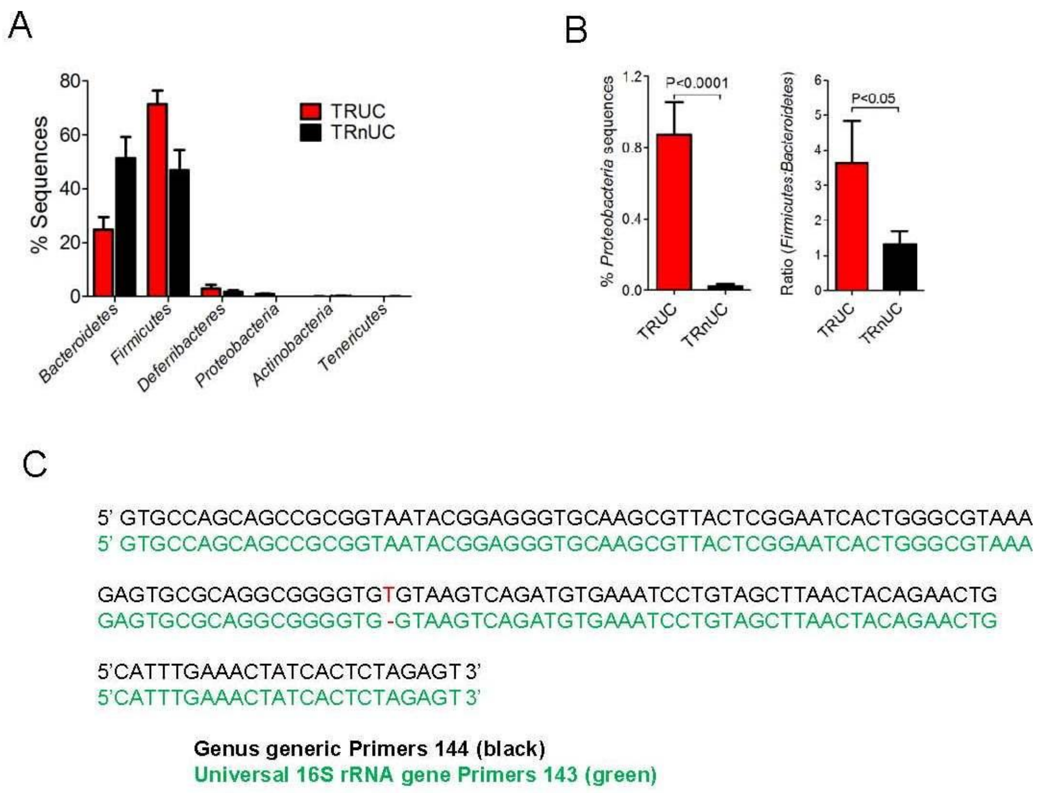

Figure S3. ***Tbx21*** $^{-/-}$ ***Rag2*** $^{-/-}$ **Mice with and without Colitis Have Distinct Intestinal Microbiota Community Profiles**

(A) Mean phylum level differences in the intestinal microbiota from TRUC (n=4) and TRnUC (n=8) mice following sequencing of bacterial 16S rRNA genes. Results show mean, and error bars represent SEM. (B) Proportion (%) of sequences from the *Proteobacteria* phylum (left panel) and the ratio of *Firmicutes* to *Bacteroidetes* (right panel) in the intestinal microbiota of TRUC (n=4) and TRnUC (n=8) mice following sequencing bacterial 16S rRNA genes. Results show mean, and error bars represent SEM. (C) 2% Agarose gel electrophoresis of a *Helicobacter* genus-generic PCR performed on a fresh fecal sample from 12 week old TRUC mouse (upper panel). The band was excised and DNA sequenced. Positive (+) and negative (-) controls are illustrated. (D) The sequence of the PCR product (black sequence) was compared with archived DNA sequences in the NCBI GenBank database and with the amplicons generated using the universal bacterial primer sets for 454 sequencing (green sequence), which confirmed that the *Helicobacter* present in the feces of TRUC mice shared >99% 16S rRNA gene sequence similarity with *H. typhlonius*. Other potentially confounding infections were excluded using the commercially available PRIA panel (Charles River Laboratories, UK) (data not shown) .

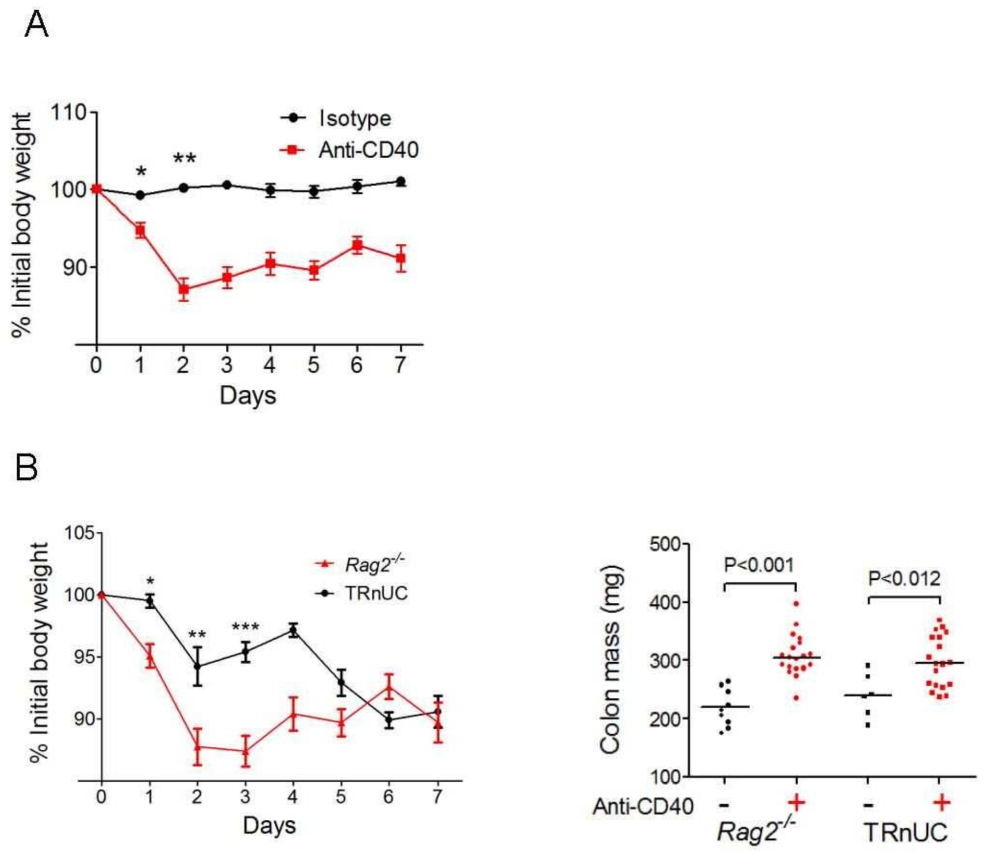

Figure S4. Agonistic CD40 mAbs Induced Wasting Disease in *Rag2*$^{-/-}$ and TRnUC Mice

(A) Weight loss (as a % on initial bodyweight) in anti-CD40 (n=7, red line) or control mAb (n=6, black line) treated mice. *P<0.007, **P<0.002. Results show mean, and error bars represent SEM. (B) Weight loss (as a % on initial bodyweight, left panel) and colon mass (right panel) in *Rag2*$^{-/-}$ and TRnuC mice following anti-CD40 treatment. *P<0.0005, **P<0.01. ***P<0.001. Results show mean, and error bars represent SEM.

Table S1. Organisms Identified in the Intestinal Microbiota of TRUC Mice that Were Absent from TRnUC Mice

**NCBI BLAST ID**	**Phylum**	**Family**	**Genus**	**p Value**
Helicobacter typhlonius	P	*Helicobacteraceae* (100)	*Helicobacter* (100)	$<2x10^{-9}$
clone SWPT20_aaa01b08 (*Parasutterella excrementihominis*, 94%)	P	*Alcaligenaceae* (100)	*Parasutterella* (100)	$<1x10^{-10}$
Parabacteroides distasonis	B	*Porphyromonadaceae* (100)	*Parabacteroides* (100)	$<1x10^{-10}$
clone mcbc135 (*Bacteroides splanchnicus*, 91%)	B	*Porphyromonadaceae* (97)	*Odoribacter* (96)	0.012
Alistipes shahii	B	*Rikenellaceae* (100)	*Alistipes* (100)	$<1x10^{-10}$
clone ncd889c07c1 (*Rikenella microfusus* , 89%)	B	unclassified	unclassified	$<1x10^{-10}$
Anaerotruncus colihominis	F	*Ruminococcaceae* (100)	*Acetanaerobacterium* (72)	$<5x10^{-10}$
clone myd3_aaa01e03, 98% (*Coprobacillus cateniformis*, 94%)	F	*Erysipelotrichaceae* (100)	*Coprobacillus* (59)	$7x10^{-5}$
clone nby399h05c1 (*Eubacterium plexicaudatum*, 93%)	F	*Lachnospiraceae* (73)	unclassified	$<6x10^{-5}$
clone nby337c02c1 (*Clostridium algidixylanolyticum*, 97%)	F	*Lachnospiraceae* (100)	Unclassified *Lachnospiraceae* (79)	<0.0004

clone LKMC046 (*Clostridium algidixylanolyticum,* 92%)	F	*Lachnospiraceae* (95)	Unclassified *Lachnospiraceae* (66)	$<3x10^{-10}$
clone HTM1039Pw-B48, 90% (*Clostridium piliforme*, 88%)	F	unclassified	unclassified	$<1x10^{-10}$

Phylum Key: P = *Proteobacteria*, B = *Bacteroidetes*, F = *Firmicutes.* Phylum, Family and Genus classifications were generated using the RDP Classifier tool (Cole et al., 2009). Figures in brackets indicate the percentage probability for each RDP classification

Table S2. Barcodes Used for Each 454-Sequenced Mouse Fecal Sample

**Sample**	**Barcode and 16S rRNA Gene Primer**
TRUC1	GACTGTGTCCGTCAATTCMTTTRAGT
TRUC2	GAGACTGACCGTCAATTCMTTTRAGT
TRUC3	GAGAGTGTCCGTCAATTCMTTTRAGT
TRUC4	GAGTAGTGCCGTCAATTCMTTTRAGT
TRnUC1	GAGTCTGTCCGTCAATTCMTTTRAGT
TRnUC2	GAGTGTGACCGTCAATTCMTTTRAGT
TRnUC3	GATCTGCACCGTCAATTCMTTTRAGT
TRnUC4	GATGTGCTCCGTCAATTCMTTTRAGT
TRnUC5	GACACTGTCCGTCAATTCMTTTRAGT
TRnUC6	GACAGTGACCGTCAATTCMTTTRAGT
TRnUC7	GACTAGAGCCGTCAATTCMTTTRAGT
TRnUC8	GACTCTGACCGTCAATTCMTTTRAGT

The primer sequences were as follows:

357F -*CTATCCCCTGTGTGCCTTGGCAGTCTCAG*ACTCCTACGGGAGGCAGCAG and 926R–**CCATCTCATCCCTGCGTGTCTCCGACTCAG**-barcode_sequence-CCGTCAATTCMTTTRAGT, where bases in italics are the 454 Lib-L ‘B’ adaptor and bases in bold are the 454 Lib-L ‘A’ adaptor.
